# Supplementary material for: Design and Proof of Concept of a Web-Based Questionnaire to Identify Patients at Risk for HIV and HCV Infection
Source: Biomedicines. 2024 Aug 14;12(8):1846. doi: 10.3390/biomedicines12081846 (PMC11352123; doi:10.3390/biomedicines12081846)
Supplement: Supplementary file 1 [file biomedicines-12-01846-s001.zip › biomedicines-3126535-supplementary.pdf]

Design and Proof of Concept of a Web-Based Questionnaire to Identify Patients at Risk for HIV and HCV Infection

Alejandro G. García-Ruiz de Morales[1,2,3], María Jesús Vivancos[1,2], Jorge Lázaro[3,4], Beatriz Romero Hernández[4,6], Beatriz Mateos[7], Pilar Pérez-Elías[8], Margarita Herrero Delgado[9], Laura Villanova Cuadra[15], Santiago Moreno Guillén[1,2,3], Javier Martínez-Sanz[1,2]*, María Jesús Pérez-Elías[1,2]*.

Supplementary Material

Table of contents

Web-based questionnaire to identify patients at risk for HIV and HCV infection at the primary care level: Inclusion criteria for Expert Panel Members. 2

List of questions and responses of the 1st Delphi round. 3

Table S1: Sexual habits 5

Table S2: Drug habits 6

Table S3: Sexually Transmitted Infections 7

Table S4: HIV Indicator Conditions 8

Section S1. Final questionnaire. 9

Section S2. Rule-based algorithm for the test and PrEP. 14

Section S3: Calculation of Questionnaire Accuracy. 16

Web-based questionnaire to identify patients at risk for HIV and HCV infection at the primary care level: Inclusion criteria for Expert Panel Members.

1. Inclusion criteria for selecting the Expert Panel members

- Recommended by any member of the investigator's group.
- Medical doctors with the desired medical specialities (internal medicine, microbiology, general medicine, gastroenterology).
- ≥5 year of experience in their specialty
- ≥10 attended patients with risk of HIV/HCV per month
- Declare no conflict of interest
- Available and willing to participate.

2. Types of experts

- Internal medicine physicians who attend HIV/HCV patients.
- Infectious diseases physicians
- Primary care physicians who treat sexually transmitted infections
- Gastroenterology physicians

List of questions and responses of the 1st Delphi round.

Domain[†]	Indicator Name	Unnecessary	Questionable	Essential
1	Sex	0/19	1/19	18/19
1	Sexual orientation	0/19	1/19	18/19
1	Level of education	1/19	5/19	13/19
1	Ethnicity	5/19	7/19	7/19
1	Country of Origin	0/19	3/19	16/19
1	Sex with transgender individuals	0/19	1/19	18/19
1	Sex worker	0/19	1/19	18/19
2	Different sexual partners	0/19	1/19	18/19
2	Serological status of recent partners	2/19	0/19	17/19
2	Any HIV positive?	2/19	1/19	16/19
2	Any HCV positive?	1/19	2/19	16/19
2	Are they your current partner?	2/19	3/19	14/19
2	If HIV +, are they virologically suppressed?	1/19	1/19	17/19
2	If HCV +, have they received treatment?	1/19	2/19	16/19
2	Unprotected sex	0/19	1/19	18/19
2	Was it in the last year?	1/19	3/19	15/19
3	Parenteral drug use	0/19	0/10	19/19
3	Methadone use	2/19	5/19	12/19
3	Slamsex ever?	0/19	1/19	18/19
3	Chemsex ever?	0/19	0/19	19/19
3	Were any of them in the last year?	0/19	0/19	19/19
4	STIs in the last year?	0/19	0/19	19/19
4	Herpes	0/19	2/19	17/19
4	Syphilis	0/19	2/19	17/19
4	Urethritis	0/19	3/19	16/19
4	Chlamydia	0/19	2/19	17/19
4	Condylomas	0/19	3/19	16/19
4	Gonorrhoea	0/19	2/19	17/19
4	Ever received post-exposure prophylaxis?	0/19	0/19	17/19
4	Previous treatment with Penicillin G benzathine	2/19	6/19	9/19
5	Accidental HIV/HCV exposition	0/19	2/19	17/19
5	Blood transfusions before 1990	0/19	3/19	16/19
6	Availability of PrEP	0/19	1/19	18/19
6	Do you want information about PrEP?	0/19	1/19	18/19
6	Do you think you could benefit of PrEP?	0/19	1/19	18/19
	Have you ever suffered of:			
7	Lymphoma	0/19	0/19	19/19
7	Anal or cervical dysplasia or cancer	0/19	0/19	19/19

7	Shingles	0/19	1/19	18/19
7	Hepatitis	0/19	0/19	19/19
7	Mononucleosis syndrome	0/19	1/19	18/19
7	Thrombo/lymphopenia	0/19	0/19	19/19
7	Extense seborrheic dermatitis	0/19	2/19	17/19
7	Prolonged and unexplained fever	0/19	0/19	19/19
7	Recurrent oral or vaginal candidiasis	0/19	0/19	19/19
7	Oral villous leucoplakia	0/19	1/19	18/19
7	Chronic (>3 months) unexplained diarrhoea	0/19	0/19	19/19
7	Unexplained weight loss	0/19	0/19	19/19
7	Tuberculosis	0/19	0/19	19/19
7	Pneumonia	0/19	1/19	18/19
8	Have you visited a medical facility recently?	0/19	2/19	17/19
8	Have you been previously tested for HIV/HCV?	0/19	1/19	18/19
8	When was your last test?	0/19	2/19	17/19
8	Have you ever donated blood?	1/19	3/19	15/19
9	Was the is simple to answer the questions?	0/19	1/19	18/19
9	Did you need help to fill the questionnaire?	2/19	1/19	16/19
9	What device did you use to complete it?	1/19	2/19	16/19
9	Did you find the tool easy to use?	3/19	2/19	14/19
9	Would you recommend it to others?	2/19	2/19	15/19

† Domains: 1) Demographic questions; 2) Sexual habits; 3) Drug use; 4) Sexually transmitted infections; 5) Accidental exposure; 6) Knowledge of PrEP; 7) HIV indicator conditions; 8) Missed opportunities; 9) Evaluation of the tool.

Table S1: Sexual habits

Participants	Attending Primary care (n=41)	With a recently acquired HIV infection (n=10)	With HIV infection acquired >6m before (n=20)	On a PrEP program (n=21)	With an active HCV infection (n=10)
**Sexual contact in the last 10 years; Yes, n (%)**	38 (92.7)	10 (100)	20 (100)	21 (100)	7 (70)
**Type of sexual contact, n (%)**					
Heterosexual	27 (65.9)	0	1 (5)	0	7 (70)
Gay and Bisexual men	10 (24.4)	10 (100)	19 (95)	21 (100)	0
**Sexual partners in the last year, n (%)**					
No	27 (65.9)	2 (20)	5 (25)	0	7 (70)
1-2	4 (9.8)	3 (30)	3 (15)	1 (4.8)	0
3-10	4 (9.8)	2 (20)	5 (25)	6 (28.6)	0
>10	1 (2.4)	3 (30)	4 (20)	14 (66.7)	0
**Use of condom, n (%)**					
Always	12 (29.3)	3 (30)	8 (40)	1 (4.7)	3 (30)
Occasionnally	9 (22.0)	4 (40)	9 (45)	17 (81.0)	2 (20)
Never	15 (36.6)	2 (20)	2 (10)	3 (14.3)	2 (20)
**Ever payed for sex, n (%)**	2 (4.9)	3 (30)	0	4 (19.1)	3 (30)

Abbreviations: HIV, human immunodefficiency virus; HCV, hepatitis C virus; PrEP, HIV pre-exposure prophylaxis

Table S2: Drug habits

Participants	Attending Primary care (n=41)	With a recently acquired HIV infection (n=10)	With HIV infection acquired >6m before (n=20)	On a PrEP program (n=21)	With an active HCV infection (n=10)
**Any drug use ever, n (%)**	4 (9.8)	2 (20)	6 (30)	8 (38.1)	6 (60)
**Chemsex ever, n (%)**	2 (4.9)	2 (20)	6 (30)	8 (38.1)	0 (0)
In the last year	2 (100)	2 (100)	6 (100)	7 (87.5)	0 (0)
**Injectable drug use ever, n (%)**	2 (4.9)	2 (20)	1 (5)	0 (0)	6 (60)
**Slamsex ever, (n %)**	1 (2.4)	1 (10)	1 (5)	0 (0)	0 (0)
In the last year	1 (100)	1 (100)	1 (100)	0 (0)	0 (0)

Abbreviations: HIV, human immunodefficiency virus; HCV, hepatitis C virus; PrEP, HIV pre-exposure prophylaxis

Table S3: Sexually Transmitted Infections

Participants	Attending Primary care (n=41)	With a recently acquired HIV infection (n=10)	With HIV infection acquired >6m before (n=20)	On a PrEP program (n=21)	With an active HCV infection (n=10)
**PEP ever, n (%)**	0 (0)	1 (10)	0 (0)	3 (14.3)	0 (0)
**STI ever, n (%)**	12 (29.3)	5 (50)	17 (85)	15 (71.4)	2 (20)
**STI in the last year, n (%)**	4 (33.3)	2 (40)	5 (29.4)	6 (40)	0
**Type of STI, n (%)**					
Genital herpes	2 (16.7)	1 (20)	3 (17.7)	3 (20)	0
Syphilis	3 (25)	1 (20)	8 (47.1)	4 (26.7)	1 (50)
Human papilloma virus	3 (25)	2 (40)	3 (17.7)	3 (20)	0
Chlamydia	2 (16.7)	1 (20)	4 (23.5)	6 (40)	0
Gonorrhoeae	4 (33.3)	2 (40)	6 (35.3)	6 (40)	1 (50)
Monkeypox	0	0	3 (17.7)	1 (6.7)	0

Abbreviations: HIV, Human immunodeficiency virus; HCV, Hepatitis C virus; PrEP, HIV pre-exposure prophylaxis; PEP, HIV post-exposure prophylaxis; STIs, sexually transmitted infections;

Table S4: HIV Indicator Conditions

Participants	Attending Primary care (n=41)	With a recently acquired HIV infection (n=10)	With HIV infection acquired >6m before (n=20)	On a PrEP program (n=21)	With an active HCV infection (n=10)
Any HIV IC, n (%)	18 (43.9)	6 (60)	13 (65)	12 (57.1)	9 (90)
Number of HIV IC, mean (SD)	0.59 (0.77)	1.2 (1.62)	1.45 (1.57)	0.86 (0.96)	2.6 (2.17)

Abbreviations: HIV, human immunodeficiency virus; HCV, hepatitis C virus; PrEP, HIV pre-exposure prophylaxis; IC, indicator conditions; SD, standard deviation

Section S1. Final questionnaire.

1. Questions in the final questionnaire [name of the variable]

1. Primary care centre.
2. Primary care physician or nurse.
3. ID number.
4. Year of birth.
5. Phone number (we will only use it to contact you if you give us explicit permission at the end of the questionnaire).
6. email address (only to receive final information from the questionnaire).
7. Current sex [sexo]:
    a. Female.
    b. Male.
    c. Trans male.
    d. Trans female.
8. Level of education [estudios]:
    a. Primary.
    b. Secondary/college.
    c. University or higher.
9. Country of origin [pais].
10. Current employment situation [situacion_labora]:
    a. Student.
    b. Active worker
    c. Unemployed.
    d. Receiving a pension.
11. Type of sexual relationships [tipo_relaciones]:
    a. Heterosexual.
    b. Homosexual.
    c. Bisexual.
12. Have you ever had sex with transexual men or women? Yes or no [relaciones_trans].
13. Have you ever paid or received money for sex? Yes or no [sexo_pagado].

14. Have you had several sexual partners in the past year? [varias_parejas].
    a. No.
    b. 1 to 2.
    c. 3 to 10.
    d. More than 10.
15. Were any of your previous partners HIV- or HCV-positive? [vih_vhc]
    a. Yes.
    b. No.
    c. I don’t know.

If the previous question was answered yes,

   d. HIV: Yes or No [vih].

   If the previous question was answered yes,

      i. Is he/she/them undetectable? [trat_controlado]

   e. HCV: yes or no. [vhc]

   If the previous question was answered yes,

      i. Is he/she/them treated and cured? [trat_curado]

   f. Is he/she/them your current partner? [pareja_actual]

16. Do you use a condom in your sexual relationships? (select the most appropriate) [preservativo].
    a. Always.
    b. Occasionally.
    c. Never.
17. Have you ever used injected drugs? Yes or no. [drogas_intra]

If the previous question was answered yes,

   a. Do you currently use them? Yes or no. [drogas_intra_actual]

18. Have you ever used methadone? Yes or no. [metadona]
19. Do you practice slamsex? Yes or no. [slamsex]

If the previous question was answered yes,

   a. Was it in the past year? Yes or no. [fecha_slamsex]

20. Do you practice chemsex? Yes or no. [chemsex]

If the previous question was answered yes,

   a. Was it in the past year? Yes or no. [fecha_chemsex]

21. Have you ever suffered from an STI? Yes or no [infeccion_sexual]:

If the previous question was answered yes,

   a. Was it in the past year? Yes or no. [fecha_infeccion]

If question 21 was answered with a yes, select all that applies.

   a. Genital Herpes (painful vesicles): yes or no. [herpes]
   b. Syphilis: yes or no. [sifilis]
   c. Human papilloma virus (HPV) or condylomes: yes or no. [papiloma]
   d. Chlamydia: yes or no. [chlamydia]
   e. Gonorrhoea: yes or no. [gonorrea]
   f. Monkeypox: yes or no. [monkeypox]

22. Do you think you could have been at risk of acquiring HIV or HCV infection via professional or accidental exposure, or via tattoos or piercings made under unhygienic conditions? Yes or no. [riesgo_condiciones]
23. Did you receive a blood transfusion before 1990? Yes or no. [transfundir]
24. Have you received post-exposure HIV prophylaxis more than once? Yes or no. [profilaxis]

If the previous question was answered yes,

   a. Was it in the past year? Yes or no. [fecha_profilaxis]

25. Have you ever heard of HIV pre-exposure prophylaxis? Yes or no. [trat_prep]
26. If you were a candidate, would you accept our phone contact to explain the possibilities of HIV and HCV prevention? Yes or no. [contacto_prep]

27. Do you think you could be a candidate, or do you wish to be included in the PrEP program? Yes or no. [si_prep]
28. If you do not think you are a candidate, would you like more information about this matter? Yes or no. [no_prep]
29. Have you ever suffered from any of these diseases? (select all that apply.)
    a. Linfoma. [linfoma]
    b. Anal or cervical dysplasia or cancer. [canceranal]
    c. Shingles. [zoster]
    d. Hepatitis (A, B or C) or an unexplained liver disease. [hepatitis]
    e. Mononucleosis syndrome after being sexually active. [mononucleosis]
    f. Thrombocytopenia or lymphopenia (low platelets or lymphocytes). [trombolinfopenia]
    g. Extense seborrheic dermatitis. [dermatitis]
    h. Unexplained fever. [fiebre]
    i. Recurrent oral or vaginal candidiasis with no antibiotics use. [candidiasis]
    j. Oral villous leucoplakia. [leucoplasia]
    k. Chronic (>3 months) unexplained diarrhoea. [diarrea]
    l. Unexplained weight loss. [perdida_peso]
    m. Tuberculosis. [tuberculosis]
    n. Pneumonia. [neumonia]
30. Did you attend any medical facility (emergency room, primary care, hospital, or work physician) in the last two years? Yes or no. [acudido_centro]
31. Have you ever been tested for HIV or HCV infection? [vih_vhc_previo]

If the previous question was answered yes,

    a. Do you remember the approximate year? [fecha_prueba]

32. Have you ever donated blood? [donar_sangre]
33. What device did you use to complete the questionnaire? [plataf_cuest]
    a. Personal mobile device.
    b. Healthcare personnel’s mobile devices
    c. Personal computer.
    d. Personal tablet.

e. A member of the staff filled out the questionnaire.

34. Did you find it simple to answer the questions? Yes or no. [cuest_sencillo]
35. Did you need help to complete the questionnaire? Yes or no. [cuest_ayuda]
36. Did you find the tool easy to use? Yes or no. [cuest_uso]
37. Would you recommend this tool to other users? Yes or no. [cuest_recomend]

2. Questions flagged neutral by DELPHI consensus.

1. Level of education: primary, Secondary/college, University, or higher.
2. Ethnicity: Black, white, Hispanic, Chinese, Indian, and others (specified).
3. Have you ever used methadone? Yes or no.
4. Have you ever received intramuscular Penicillin G treatment? Yes or no.

3. Deleted questions after DELPHI consensus.

1. Ethnicity: Black, white, Hispanic, Chinese, Indian, and others (specified).
2. Have you ever received intramuscular Penicillin G treatment? Yes or no.

Section S2. Rule-based algorithm for the test and PrEP.

1. Need for a test:

Calculated with the following code: @HIDDEN-SURVEY @CALCTEXT(if(([tipo_relaciones] = '1' and [varias_parejas] = '2') or ([tipo_relaciones] = '1' and [varias_parejas] = '3') or ([tipo_relaciones] = '2' and [varias_parejas] = '2') or ([tipo_relaciones] = '2' and [varias_parejas] = '3') or ([tipo_relaciones] = '1' and [infeccion_sexual] = '1') or ([tipo_relaciones] = '1' and [infeccion_sexual] = '2') or ([tipo_relaciones] = '2' and [infeccion_sexual] = '1') or ([tipo_relaciones] = '2' and [infeccion_sexual] = '2') or ([relaciones_trans] = '1') or ([sexo_pagado] = '1') or ([varias_parejas] = '2' and [preservativo] = '1') or ([varias_parejas] = '2' and [preservativo] = '2') or ([varias_parejas] = '3' and [preservativo] = '1') or ([varias_parejas] = '3' and [preservativo] = '2') or ([vih_vhc] = '1' and [vih_vhc_previo] = '0') or ([pareja_actual] = '1') or ([drogas_intra] = '1') or ([slamsex] = '1') or ([chemsex] = '1') or ([infeccion_sexual] = '1') or ([profilaxis] = '1') or ([riesgo_condiciones] = '1' and [vih_vhc_previo] = '0') or ([transfundir] = '1' and [vih_vhc_previo] = '0') or ([linfoma] = '1' or [canceranal] ='1' or [zoster] ='1' or [hepatitis] ='1' or [mononucleosis] ='1' or [trombolinfopenia] ='1' or [dermatitis] ='1' or [fiebre] ='1' or [candidiasis] ='1' or [leucoplasia] ='1' or [diarrea] ='1' or [perdida_peso] ='1' or [tuberculosis] ='1' or [neumonia] ='1'),'Con las respuestas registradas le ofrecemos la posibilidad de realizarse un test de VIH de MANERA GRATUITA en su centro de Salud. En caso de no estar con su médico en el momento actual, notifíquese el resultado a su médico y/o enfermera de su centro de salud, que le darán las indicaciones para realizarse dicha prueba SIN NINGÚN COSTE para Ud.','NO es necesario la realización de un test directo'))

2. Need for PrEP.

Calculated with the following code: @HIDDEN-SURVEY @CALCTEXT(if([sexo_pagado]=1 and [preservativo]>0,'SI', if(([sexo]>0 and [tipo_relaciones]>0) and (([varias_parejas]=3 and [preservativo]>0) or ([varias_parejas]=3 and ([fecha_slamsex]=1 or [fecha_chemsex]=1)) or ([varias_parejas]=3 and [profilaxis]=1) or ([varias_parejas]=3 and [infeccion_sexual]=1) or ([preservativo]>0 and ([fecha_slamsex]=1 or [fecha_chemsex]=1)) or ([preservativo]>0 and [profilaxis]=1) or ([preservativo]>0 and [infeccion_sexual]=1) or (([fecha_slamsex]=1 or [fecha_chemsex]=1) and [profilaxis]=1) or (([fecha_slamsex]=1 or [fecha_chemsex]=1) and [infeccion_sexual]=1) or ([profilaxis]=1 and [infeccion_sexual]=1)),'SI', if((([sexo]< 2 and [tipo_relaciones]='0') or [drogas_intra_actual]=1 or [metadona]=1) and [preservativo]>0 and (([varias_parejas]=3 and ([fecha_slamsex]=1 or [fecha_chemsex]=1)) or ([varias_parejas]=3 and [profilaxis]=1) or ([varias_parejas]=3 and [infeccion_sexual]=1) or (([fecha_slamsex]=1 or [fecha_chemsex]=1) and [profilaxis]=1) or (([fecha_slamsex]=1 or [fecha_chemsex]=1) and [infeccion_sexual]=1) or ([profilaxis]=1 and [infeccion_sexual]=1)),'SI', if(([sexo]>0 and [tipo_relaciones]>0) and ([varias_parejas]=3 or [preservativo]>0 or ([fecha_slamsex]=1 or [fecha_chemsex]=1) or [profilaxis]=1 or [infeccion_sexual]=1),'REVISIÓN', if((([sexo]< 2 and [tipo_relaciones]='0') or [drogas_intra_actual]=1 or

[metadona]=1) and [preservativo]>0 and ([varias_parejas]=3 or ([fecha_slamsex]=1 or [fecha_chemsex]=1) or [profilaxis]=1 or [infeccion_sexual]=1),'REVISIÓN','NO'))))))

Section S3: Calculation of Questionnaire Accuracy.

For HIV/HCV Testing: True Positives (TP): 61 out of 62; False Negatives (FN): 1 (missed HIV on follow up); False Positives (FP): 2 (assumed as an estimation); True Negatives (TN): 38 (total participants - TP - FN - FP).

Sensitivity: $\frac{61}{62} \approx 0.984$

Specificity: $\frac{38}{40} = 0.950$

Accuracy: $\frac{61+38}{61+38+2+1} = \frac{99}{102} \approx 0.971$

For PrEP Derivation: True Positives (TP): 48 out of 51; False Negatives (FN): 3; False Positives (FP): 2 (assumption as an estimation); True Negatives (TN): 49 (total participants - TP - FN - FP)

Sensitivity: $\frac{48}{51} \approx 0.941$

Specificity: $\frac{49}{51} \approx 0.961$

Accuracy: $\frac{48+49}{48+49+2+3} = \frac{97}{102} \approx 0.951$

To calculate these values, we had to make some assumptions based on the current HIV prevalence in Spain and our previous studies. Given that the current HIV prevalence in Spain is less than 0.25%, according to the Spanish Ministry of Health, and that in our earlier study, DRIVE-03, we encountered an even lower prevalence in our area, we have assumed that the false negative for HIV and HCV testing is only 1, individual, which is the one that was missed by the questionnaire from the "HIV infection acquired >6m before". We also assumed that the number of false positives in the questionnaire was two, which is probably overestimated because the main objective was to identify as many patients as possible subject to testing. For PrEP derivation calculations, we assumed three false negatives: those who were not flagged for PrEP evaluation even though they were HIV positive (although diagnosed more than six months before, they might no longer have been at risk at the time of the questionnaire). We assumed two false positives; however, false positives in this part of the questionnaire would not be an issue, as the questionnaire attempts to be very sensitive to prevent new infections in as many patients as possible.
